# After Extraction, Upper Premolars Undergo Programmed Socket Collapse with Development of Cavitations Rather than Complete Socket Healing: A Radiological Study

**DOI:** 10.3390/bioengineering12020128

**Published:** 2025-01-29

**Authors:** Shahram Ghanaati, Joanna Śmieszek-Wilczewska, Sarah Al-Maawi, Anja Heselich, Robert Sader

**Affiliations:** 1 FORM-Lab (Frankfurt Orofacial Regenerative Medicine-Research Laboratory), Department for Oral, Cranio-Maxillofacial and Facial Plastic Surgery, Medical Center of the Goethe University Frankfurt, Goethe University, 60590 Frankfurt am Main, Germany; 2 Denticus Clinic, Lelewela 1/1, 44-100 Gliwice, Poland

**Keywords:** extraction socket healing, bone regeneration, socket preservation, implantology

## Abstract

The alveolar ridge undergoes a loss in volume and atrophy after tooth extraction. Understanding the wound healing and bone regeneration process after tooth extraction is a key factor in the insertion of dental implants. Therefore, the aim of the present study was to analyze the socket healing process after the extraction of upper premolars based on cone beam computed tomography (CBCT) over six months. Special focus was placed on the morphological changes in the alveolar crest and within the socket. A retrospective analysis of patients in need of tooth extraction in the upper premolar region was performed in this study. All patients received flapless tooth extraction under local anesthesia and CBCT immediately after tooth extraction. Further CBCT analysis was performed after three months for the first group (*n* = 18) and after six months for the second group (*n* = 18). The results showed that all sockets underwent an inward movement of the defect walls towards the defect center, resulting in reduced total alveolar ridge volume and defect volume. This result was observed after three months and persisted after six months. The inward movement was quantified as a vertical socket collapse of up to 30.1 ± 9.0% after three months and 34.3 ± 6.7% after six months. The horizontal inward movement was quantified as a buccal socket collapse of 47.7 ± 12.3% after three months and 55.7 ± 29.1% after six months. New bone formation within the socket was evident, especially in the occlusal part of the socket. Additionally, bone formation was primarily observed as bone apposition along the socket walls and did not reach the defect center in most cases. The combination of socket collapse and bone apposition led to the formation of cavitations inside the socket that were mostly localized under the occlusal part. These novel findings with respect to socket collapse and formation of cavitation represent a paradigm shift and call for reconsidering the current understanding of socket healing. Based on the data, socket healing should be understood as a patient-specific process that requires 3D radiographic analysis for planning dental implants.

## 1. Introduction

Tooth loss is one of the most common events in a human lifespan. According to the World Health Organization, one in three (31.3%) adults aged 60 years or older in the European Region suffer from complete tooth loss [1]. Tooth loss has been shown to be associated with impaired quality of life [2], reflecting the importance of tooth restoration. Different techniques are available to replace missing teeth, among which dental implants are the most effective [3]. To achieve a successful restoration using dental implants, sufficient bone quality and quantity is needed.

However, after tooth extraction, a physiological remodeling process occurs during socket healing that results in general bone atrophy. One of the key factors responsible for this phenomenon is the absence of biomechanical stimulation [4]. Socket healing is a highly complex procedure as tooth extraction results in bony defects as well as injury to the soft tissues. The regeneration process in this case is influenced by multiple factors, including the regenerative potential of the patient, the type of tooth extraction, the treatment of the socket, and many more [5]. Current knowledge of socket healing shows that the volume reduction in the jaw varies between individual patients according to the tooth position, the presence of infection, previous periodontal diseases, and the extent of trauma occurring during tooth extraction [6]. In general, it has been observed that the bucco-lingual dimension of the alveolar ridge undergoes a higher resorption (loss of horizontal dimension) than the height (vertical dimension). The horizontal dimension loss reaches a mean of 32% of the original dimension after 3 months and up to 63% after 6 months, whereas the vertical dimension loss was found to be about 15% after 3 months and up to 22% after 6 months [7]. Additionally, it was shown that the atrophy of the jaw is not only limited to the alveolus healing but continues beyond that, especially in edentulous jaws [8]. Different studies attempt to outline the pathophysiological mechanism of ridge resorption to prevent jaw atrophy based on biological rational methods [7]. However, this process is still not yet fully understood.

Meanwhile, various protocols are available to treat the extraction sockets and reconstruct the atrophic jaw to allow dental implant insertion [9]. Most recent and ongoing research accepts the phenomenon of atrophy as a physiological process and focuses on the biomaterial-based regeneration of the atrophic socket or the comparison of treated to untreated sockets with the aim of generating the best possible situation for implant placement [10,11]. At the same time, there is an acute need to further understand the physiological process of socket healing in order to develop improved protocols to support bone healing and prevent atrophy.

Therefore, the present study aimed to analyze the socket healing process after the extraction of upper premolars based on cone beam computed tomography (CBCT) over 6 months. A retrospective analysis of CBCT imaging, performed directly after tooth extraction, then again after 3 and 6 months, was conducted to compare the progress of bone healing and remodeling. Special focus was placed on the morphological changes in the alveolar crest and within the socket.

## 2. Material and Methods

### 2.1. Study Design

#### 2.1.1. Study Design and Institutional Review Board (IRB) Approval

This retrospective study evaluated radiological data of patient treated at the private office of JS-W in Katowice, Poland, under the supervision of the first author SG. IRB approval (#16/2023) for this study was granted by the Bioethics committee at the Silesian Medical Chamber in Katowice, Poland (24 July 2023). The available radiological data were analyzed retrospectively using a novel method to evaluate the three-dimensional bone change as described below.

#### 2.1.2. Sample Size Calculation

An online tool https://clincalc.com/stats/samplesize.aspx?example (accessed on 15 January 2025) was utilized to perform statistical power analysis and estimate the required sample size per group. Following established standards for statistical power and significance, the parameters were set as follows: a Beta error of 20% (corresponding to a statistical power of 80%) and an alpha error of 5%. The primary endpoint of this study—new bone formation—was used to estimate the expected minimum detectable difference between the study group (3 months) and the control group (6 months). This estimation was based on radiological data from previous studies. The analysis indicated that a minimum of 18 patients per group would be necessary to achieve adequate statistical power. Consequently, a total of 36 patients (*n* = 18 per group) were included in this study.

#### 2.1.3. Inclusion and Exclusion Criteria

Patients indicated for extraction of hopeless premolars followed by implant placement using a two-stage approach, that received CBCT after tooth extraction and after 3 or 6 months were included.

The exclusion criteria were metabolic disorders, acute and untreated periodontal disease, periapical lesions, root fracture of the tooth requiring extraction, and a previously compromised alveolar crest at the defect area. Patients at risk of bisphosphonate-associated osteonecrosis, pregnant patients, patients with insufficient hygiene status, and potentially non-compliant patients were also excluded.

#### 2.1.4. Treatment and Radiological Evaluation

Tooth extraction was performed by atraumatic and flapless tooth extraction under local anesthesia. Post-extraction, the alveoli were excochleated, rinsed with sterile saline solution, and subjected to a tension-free approximation of the wound borders. CBCT images of the defect areas were taken directly after extraction and after a healing period of either three or six months.

### 2.2. Image Analysis-Based Evaluation of Bone Regeneration

The qualitative image analysis of bone regeneration after tooth extraction was based on CBCT images taken after extraction and after a defined bone regeneration period of three or six months. The images were processed using OsiriX MD (Pixmeo SARL, software version UDI-PI:14.1.1, Bernex, Switzerland) and Photoshop (Adobe Systems Software Version 26.3.0, Dublin, Ireland). CBCT data were processed in OsiriX MD (Pixmeo SARL, software version UDI-PI:14.1.1, Bernex, Switzerland) using a three-dimensional (*3D*) Surface Rendering tool to obtain 3D images of the related jaw, including the region of interest (ROI) where the tooth had been extracted. Here, the threshold was set to allow analyzing the low-mineralized as well as mature bone. The resultant 3D images containing rendering artifacts due to noise artifacts from the original CBCT image were post-processed using the software’s Meshmixer tool (Version 3.5.474), thereby achieving artifact-free 3D images for further processing.

Sectioned 3D images presenting buccal, lingual, mesial, or distal views of the ROI were created using the Meshmixer tool. Thus, hemi-sections of the extraction sockets were prepared in all four orientations at the time points immediately after extraction and after three and six months of regeneration. The images were further processed as described below.

Image-analysis-based qualitative evaluation of bone regeneration was conducted in Photoshop. For each orientation (buccal, lingual, mesial, distal), the post-extraction and post-regeneration images of ROI were overlayed.

Pseudo-color coding of the structures was used to differentiate between the structures and changes due to regeneration. First, the structures were separately differentiated at each time point. In the post-extraction images, regions of residual bone structures and, if applicable, neighboring tooth/teeth or implants were defined. An individual Layer of this area was created and colored blue.

The cavity of the extraction socket was defined accordingly, and an individual Layer was created and colored red. The post-regeneration images were processed with neighboring structures colored blue and residual cavities red, each in an individual Layer.

Different ROI masks were used for the red-marked extraction cavity, the post-extraction socket in green, the post-regeneration images with surrounding structures in blue, and the residual cavity structures in red. The resulting image showed the green-marked original size of the extraction cavity surrounded by neighboring structures with the residual cavity structures after the regeneration time placed on top, thereby visualizing the areas where the bone had been regenerated.

Bone height changes were marked by defining the alveolar crest surface line above (buccal/lingual view) or lateral/contralateral to (distal/mesial view) the post-extraction alveolus, and further transferring this surface line to the post-regeneration image showing the region of bone regeneration as described above. In this way, the newly regenerated bone structures were identified, and regions of bone loss below/lateral to the surface line of the original alveolar crest rim after extraction were also viewed by pseudo-coloring.

The final overlay images in the buccal, lingual, mesial, and distal views were used for qualitative evaluation and semi-quantitative analysis of bone regeneration and bone dimensional loss (height/width) in each orientation.

### 2.3. Statistical Analysis

Overlay images presenting the central section of the alveoli in different orientations, as described in Section 2.2, were used for semi-quantitative analysis to compare individual structures within and between groups. Occlusal loss of height in the mesiodistal and bucco-lingual orientation, lateral bone loss in the occlusal view, and lingual and buccal collapse of bone structures were determined. Based on the voxel data from the original CBCT images, the area from the overlay images of each orientation was measured in mm^2^. To directly compare any changes, the relative areas of the individual structures (bone-structure-free cavities within the alveoli, newly formed bone, collapse of the alveoli) were then calculated for each case and endpoint. Finally, the mean values ± standard deviations were calculated for each time point. Statistical analysis was performed using a two-way analysis of variance, at an alpha of 0.05, paired with a post-hoc test for multiple comparisons (uncorrected Fishers’ Least Significant Difference test) using GraphPad Prism statistical software (version 10.1.1).

## 3. Results

### 3.1. Patients

Eighteen patients were enrolled per group (3 months or 6 months of healing period), (Table 1). The initial healing was uneventful in all subjects. No acute or chronic infections were documented at any time point. The present study focused on the detailed 3D analysis of the radiological data, which is presented in the following sections.

### 3.2. Qualitative Assessment of Alveolar Closure After Three Months

The process of alveolar closure after three months showed interesting morphology that is described according to the analysis aspect as following.

**Figure 1:**
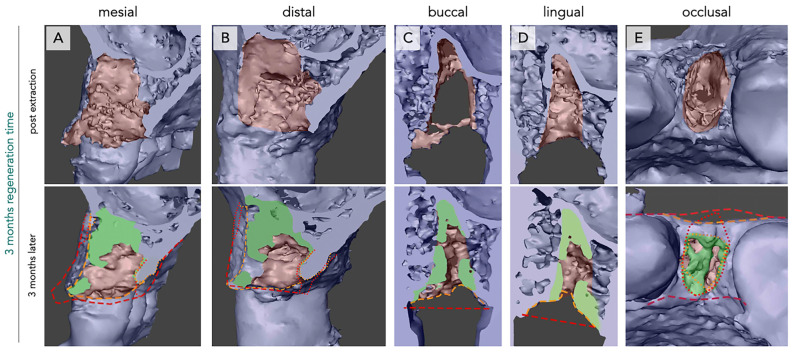
Bone regeneration and morphological changes within the premolar extraction alveolus of one rooted tooth after three months of regeneration time. Radiological imaging of extraction alveoli using CBCT directly after extraction (**upper row**) and after three months of regeneration (**lower row**) in mesial (**A**), distal (**B**), buccal (**C**), lingual (**D**), and occlusal (**E**) orientations. New bone formation (green) and differences in alveolar crest dimensions between both time points (red = post-extraction, orange = after three months) can be seen. Formation of new bone is observed apically and beginning from the socket walls, accompanied by lateral (**A**,**B**) and vertical (**C**,**D**) bone loss. The apical bone formation is predominantly seen in the buccal aspect (**A**,**E**).

**Figure 2:**
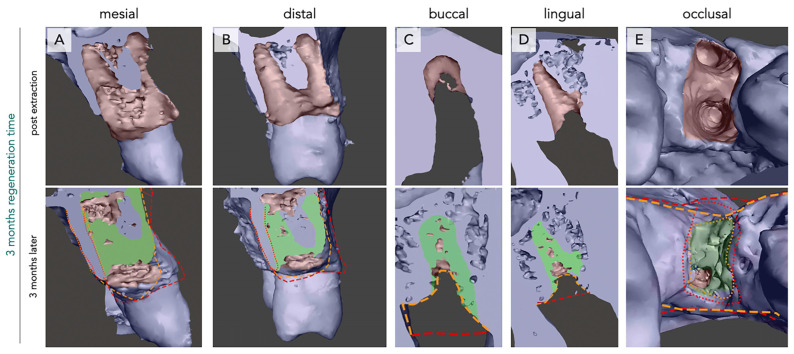
Bone regeneration and morphological changes within the premolar extraction alveolus of two rooted tooth after three months of regeneration time. Radiological imaging of the extraction alveoli using CBCT directly after extraction (**upper row**) and after three months of regeneration (**lower row**) in mesial (**A**), distal (**B**), buccal (**C**), lingual (**D**), and occlusal (**E**) orientations. New bone formation (green) and differences in alveolar crest dimensions between both time points (red = post-extraction, orange = after three months) are seen. Regeneration of bone is observed in both former root cavities; however, an apical cavity where no bone regeneration occurred is still visible (**A**,**B**). Loss of bone height is especially emphasized in the buccal perspective (**C**).

The mesio-buccal, mesio-lingual, disto-buccal, and disto-lingual lamellae seemed to undergo an inward movement towards the central part of the defect. This phenomenon was observed for all analyzed sockets and was evidenced by the difference between the position of the alveolar crest immediately after tooth extraction and after 3 months (Figure 1 and Figure 2, red and orange markings).

New bone formation within the defect showed different intra-individual patterns after 3 months. In 37.5% of the analyzed defects, no new bone formation was observed along the longitudinal axis of the defect. Twenty-five percent of the analyzed defects showed new bone formation along the defect walls, but not in the central part of the defect. In the other 37.5% of the analyzed defects, new bone formation was evident throughout the defect (Figure 1 and Figure 2C,D).

When analyzing the apical region of the defect, in direct approximation to the sinus cavity, a reduction in the sinus floor thickness was evident in all analyzed subjects (Figure 1 and Figure 2).

The occlusal part of the defect showed a different pattern. Here, again, a bucco-lingual inward movement towards the center of the defect was observed, resulting in a loss of volume in the lateral aspect. Additionally, a vertical inward movement of the crestal part was observed. In this case, the mesial and distal walls seemed to move towards the center of the defect (Figure 1E and Figure 2E).

New bone formation was observed near the defect walls, but not in the center. In general, in 87,5% of the analyzed defects, complete occlusal bone closure was not observed. Whereas, in 12,5% of the subjects, the occlusal defect part was completely closed by new bone formation (Figure 1 and Figure 2).

In general, one-rooted (Figure 1) and two-rooted (Figure 2) teeth showed similar patterns. However, new bone formation seemed to be greater in the two-rooted teeth.

### 3.3. Qualitative Assessment of Alveolar Closure After Six Months

The process of alveolar closure after six months showed several interesting phenomena, as described below.

**Figure 3:**
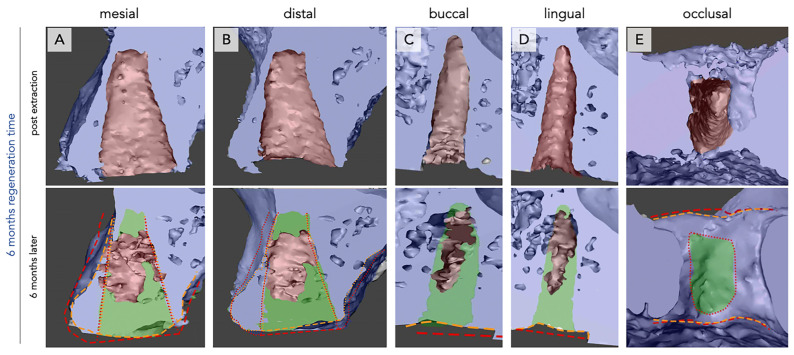
Bone regeneration and morphological changes within the premolar extraction alveolus of one rooted tooth after six months of regeneration time. Radiological imaging of the extraction alveoli using CBCT performed directly after extraction (**upper row**) and after six months of regeneration (**lower row**) in mesial (**A**), distal (**B**), buccal (**C**), lingual (**D**), and occlusal (**E**) orientations. New bone formation (green) and differences in alveolar crest dimensions between both time points (red = post-extraction, orange = after six months) are seen. Bone regeneration is observed to a large extent occlusally and to lower extent apically, leaving a non-regenerated empty region in the center of the alveoli (**A**–**D**). Mild lateral or vertical bone loss is seen (**A**–**E**).

**Figure 4:**
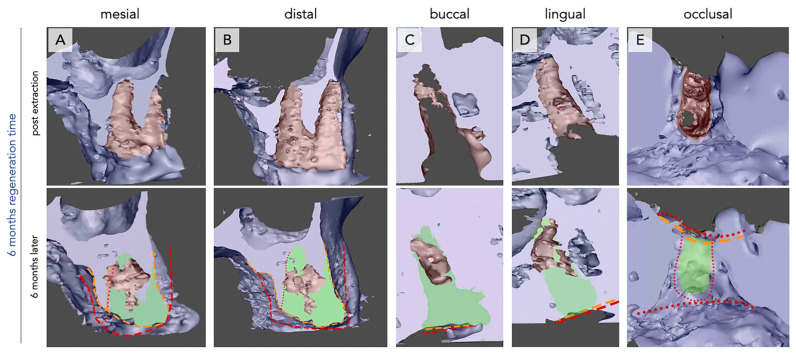
Bone regeneration and morphological changes within the premolar extraction alveolus of two rooted tooth after six months regeneration time, case #6. Radiological imaging of the extraction alveoli using CBCT performed directly after extraction (**upper row**) and after six months of regeneration (**lower row**) in mesial (**A**), distal (**B**), buccal (**C**), lingual (**D**), and occlusal (**E**) orientations. New bone formation (green) and differences in alveolar crest dimensions between both time points (red = post-extraction, orange = after six months) are seen. Most of the socket shows bone regeneration, but more bone formation is seen in the occlusal region. A cavity is seen in the apical part of the socket without any new bone formation (**A**–**E**). Partial bone atrophy is observed as well, mainly laterally (**A**,**B**).

The mesio-buccal, mesio-lingual, disto-buccal, and disto-lingual lamellae each showed a pronounced inward movement towards the central part of the defect. This phenomenon was observed for all analyzed subjects (Figure 3 and Figure 4).

After six months, new bone formation showed different intra-individual patterns. Twenty-five percent of the analyzed defects showed new bone formation in direct approximation to the defect walls, but the bone did not reach the center. In 37.5% of the analyzed defects, total closure of the defect by new bone formation was only observed in the crestal part. A further 37.5% showed new bone formation throughout the defect (Figure 3 and Figure 4).

In the apical part of the defect, further pronounced loss of the bone thickness of the sinus floor was observed (Figure 3 and Figure 4).

The occlusal aspect of the defect showed a further inward movement of the defect walls (bucco-lingual and mesiodistal) after six months. This movement seemed to further reduce the diameter of the defect. Compared with the findings after three months, those after six months revealed that the inward movement of the bucco-lingual dimension contributed to volume loss of the lateral aspect of the alveolus (Figure 3 and Figure 4, red vs. orange markings). The observed alveolar closure mechanism led to reduced socket volume, for both vertical and horizontal aspects (Figure 3 and Figure 4). It seems that occlusal ossification could mature within a period of six months, regardless of the nonmineralized tissue at the bottom of the socket.

Additionally, the regions that were not yet filled with new bone formation turned into cavitation when the socket underwent the observed inward movement.

In general, one-rooted (Figure 3) and two-rooted (Figure 4) teeth showed similar patterns. However, new bone formation seemed to be greater in the two-rooted teeth.

### 3.4. Quantitative Analysis of the Non-Ossified Area, New Bone Formation, and Vertical Bone Loss Within the Alveolus from the Bucco-Lingual Perspective: At Three Months vs. Six Months After Extraction

**Figure 5:**
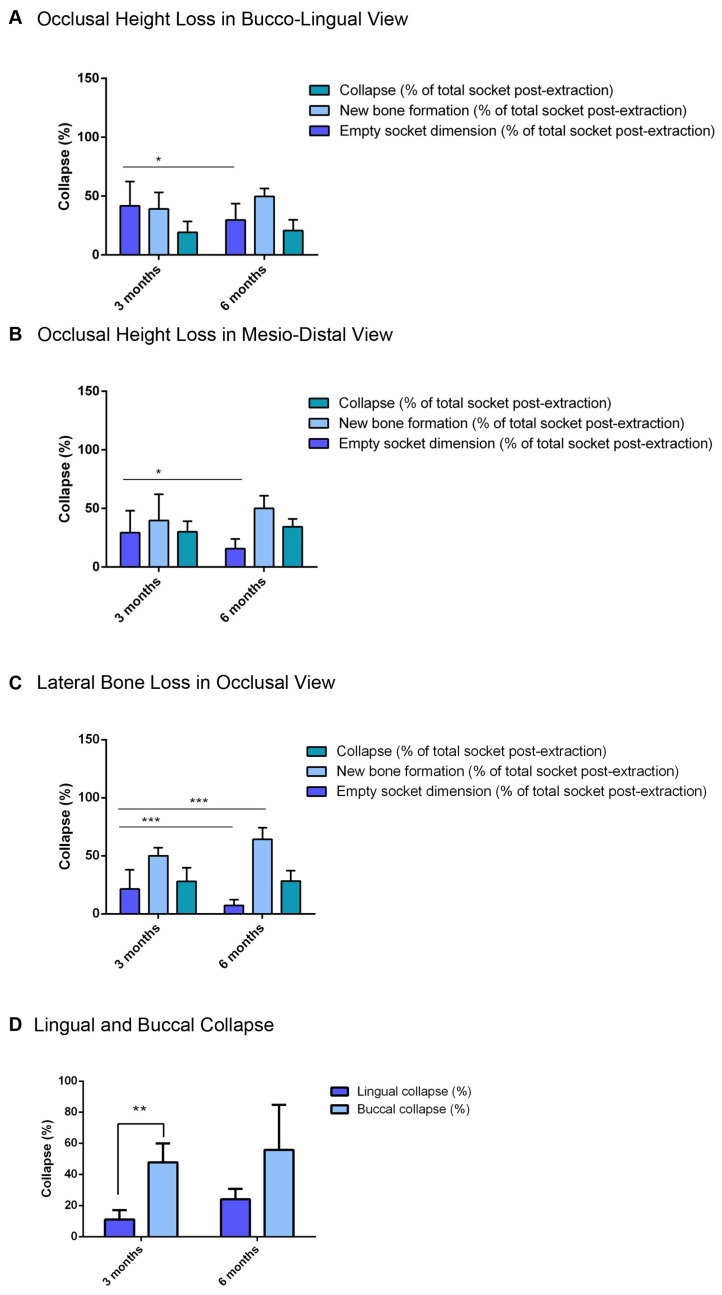
(**A**) Quantitative analysis in the bucco-lingual view. Relative volume (%) of “new bone formation”, “empty socket dimension”, and “collapse” measured in the bucco-lingual view in relation to the initial extraction socket volume for sockets with three and six months of regeneration time (*n* = 18 each). No significant differences in the three parameters are observed among the analyzed groups. (**B**) Quantitative analysis in the mesiodistal view. Relative volume (%) of “new bone formation”, “empty socket dimension”, and “collapse”, measured in the mesiodistal view in relation to the initial extraction socket volume for sockets with three and six months of regeneration time. A tendency of more bone formation is seen in the 6-month group, but no significant differences in the three parameters are observed among the analyzed groups. (**C**) Lateral bone loss in the occlusal view. Relative volume (%) of “new bone formation”, “empty socket dimension”, and “collapse”, measured in the occlusal view in relation to the initial extraction socket volume for sockets with three and six months of regeneration time. A tendency of more bone formation is observed in the 6-month group; however, no significant differences in the three parameters are observed among the analyzed groups. (**D**) Collapse of the lingual and buccal portions of the socket. Relative values (%) of lingual and buccal collapse calculated in relation to the initial status for sockets after either three or six months of regeneration time. No statistically significant differences are observed between the 3- and 6-month groups for lingual or buccal collapse. However, a statistically significant effect is observed between the buccal and lingual collapse for the 3-month group, with significantly higher collapse buccally than lingually. Higher buccal values are observed in the 6-month group as well but are not statistically significant. Data are represented as means ± standard deviation (SD). * *p* < 0.05, ** *p* < 0.01, *** *p* < 0.001.

Quantitative analysis from the bucco-lingual perspective of the alveoli after three months revealed the following: 41.6 ± 20.6% for the non-ossified area, 39.1 ± 13.9% for new bone formation within the alveolus, and 19.2 ± 9.2% for the vertical alveolar collapse (Figure 5A). Quantitative analysis from the bucco-lingual perspective of the alveoli after six months revealed 29.6 ± 13.9% for the non-ossified area, 49.6 ± 6.7% for new bone formation within the alveoli, and 20.6 ± 9.2% for the vertical alveolar collapse. A statistically significant difference was only recorded for the non-ossified area (* *p* < 0.05) (Figure 5A, Table 2).

### 3.5. Quantitative Analysis of the Non-Ossified Area, New Bone Formation, and Vertical Bone Loss Within the Alveolus from the Mesiodistal Perspective: At Three Months vs. Six Months After Extraction

From the mesiodistal perspective of the alveoli after three months, the values measured were 29.3 ± 18.7% for the non-ossified area, 39.6 ± 22.3% for new bone formation within the alveolus, and 30.0 ± 9.0% for the vertical alveolar collapse (Figure 5B). In the mesiodistal perspective, the alveoli after six months had measured values of 15.6 ± 8.3% for the non-ossified area, 50.0 ± 10.7% for new bone formation within the alveoli, and 34.3 ± 6.6% for the vertical alveolar collapse (Figure 5B). A statistically significant difference was only recorded for the non-ossified area (* *p* < 0.05) (Figure 5B, Table 2).

### 3.6. Quantitative Analysis of the Non-Ossified Area, New Bone Formation, and Vertical Bone Loss Within the Alveolus from the Occlusal Perspective: At Three Months vs. Six Months After Extraction

From the occlusal perspective, the alveoli after three months had measured values of 21.4 ± 16.5% for the non-ossified area, 50.1 ± 6.9% for new bone formation within the alveolus, and 28.0 ± 11.8% for the vertical alveolar collapse (Figure 5C). Quantitative analysis from the occlusal perspective of the alveoli after six months revealed the following: 7.3 ± 4.9% for the non-ossified area, 64.3 ± 9.9% for new bone formation within the alveoli, and 28.3 ± 8.9% for the vertical alveolar collapse. Over time, the non-ossified area showed a statistically significantly lower percentage after 6 months in comparison to 3 months (*** *p* < 0.001). Similarly, a statistically significantly higher percentage of new bone formation was measured after 6 months in comparison to 3 months (*** *p* < 0.001) (Figure 5C, Table 2).

### 3.7. Quantitative Assessment of Buccal vs. Lingual Bone Lamella Collapse Three Months After Extraction

Quantitative assessment of the buccal and lingual lamellar bone collapse after three months showed a collapse of the alveolar walls of 11.0 ± 6.0% lingually and a statistically significant higher collapse of 47.6 ± 12.2% buccally (*p* < 0.01) (Figure 5D). Accordingly, the buccal lamella experienced a more pronounced collapse in the first three months compared with the lingual alveolar wall within the same time frame (Figure 5D, Table 3).

### 3.8. Quantitative Assessment of Buccal vs. Lingual Bone Lamella Collapse Six Months After Extraction

Quantitative assessment of buccal and lingual lamellar bone collapse after six months showed collapse of the alveolar walls by 24.0 ± 6.7% lingually and 55.6 ± 29.1% buccally (Figure 5D, Table 3). However, these differences were not statistically significant.

## 4. Discussion

The healing process of the alveolus defect after tooth extraction has been of high interest to clinicians and researchers for many years due to its crucial clinical role when it comes to the replacement of missing teeth with dental implants [12]. The insertion of dental implants requires biologically active and biomechanically stable bone to allow sufficient primary stability and subsequent osseointegration. Early studies that analyzed socket healing described the mechanisms of defect regeneration using different methods, especially histology in preclinical research [13].

**Figure 6:**
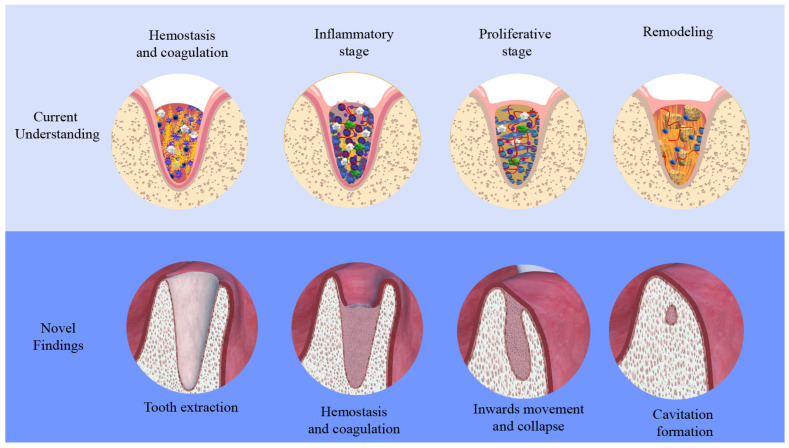
Current understanding of socket healing and the bone regeneration process within the extraction socket after tooth extraction [14] in comparison to the novel findings of this study.

The current understanding of socket healing describes it as a physiological process that includes different overlapping phases. In the first phase, the socket is filled with blood from the extraction site and inflammatory cells migrate into the blood clot (inflammation). The next step involves callus formation in the form of woven bone (proliferation). Finally, the woven bone is remodeled to mineralized lamellar bone (remodeling). In this phase, bone resorption is evidenced, leading to a dimensional change in the alveolar ridge (Figure 6) [14]. Moreover, morphological studies have shown that the alveolar volume decreases with time after tooth extraction, resulting in a loss of up to 32% of the original dimension after 3 months and up to 63% after 6 months. This dimension loss is often referred to as atrophy or bone resorption [15]. However, further understanding of the process of alveolar dimension change is still needed.

Therefore, the present study aimed to analyze the unassisted socket healing process after the extraction of upper premolars based on cone beam computed tomography (CBCT) at two time points, 3 and 6 months after extraction.

The results presented here allow further the understanding of two critical aspects of socket healing: (a) the dimensional change over time and (b) new bone formation and regeneration within the defect.

In terms of dimensional change, our results showed that an inward movement of the defect walls occurs after 3 months. It takes place from the bucco-lingual, the medio-distal, and the vertical direction towards the defect center. These inward movements were evident in all examined subjects after 3 months and persisted after 6 months. This phenomenon results in a collapse of the alveolar socket and a decrease in the defect volume, but also a decrease in the total alveolar ridge volume. The quantitative results showed a vertical alveolar collapse of up to 34.3 ± 6.7% and a horizontal alveolar collapse of up to 55.7 ± 29.1% after 6 months. The data are in alignment with currently reported data from a systematic review based on randomized controlled clinical studies that refers to a vertical dimensional change of 11–22% and a horizontal dimensional change of 29–63% at 6–7 months [16]. It is important to note that our results also show a wide range of interindividual differences. These differences may have been influenced by many parameters such as the tooth condition before extraction, the tooth position, the health of neighboring teeth or general periodontal diseases. To reduce this influence, we focused on the upper premolar and excluded patients with active untreated periodontal diseases. However, even chronic and treated periodontal changes may have influenced the socket healing leading to different patterns within the presented individuals.

The observed dimensional change in the alveolar bone was considered as a resorption process in recent studies [7,16], whereas our study is the first to describe the inward movement of the defect wall and the collapse of the socket. It seems as if this movement is the human body’s attempt to minimize the size of the socket and support the closure of the bone defect.

The second aspect is the new bone formation inside the socket defect. Our results showed two patterns of new bone formation when looking at the occlusal part compared to the rest of the socket. New bone formation was observed in direct contact with the defect walls and did not reach the defect center in most cases after 3 months. Similarly, after 6 months, the defects were not fully filled with newly formed bone. In terms of the occlusal part, new bone formation was evident after 3 months and a more mature bone was observed after 6 months. These findings show a completely different pattern of new bone formation than that described in the literature up until now. The current understanding of new bone formation inside the socket relies on the histologic results of preclinical studies that described new bone formation within the alveolar socket. In that case, new bone formation was observed as woven bone after 14 days, which was changed to mineralized bone after 30 days [13].

By contrast, our radiological findings clearly showed that new bone formation starts from the periphery and continues towards the center. These observations do not comply with the theory that sockets are filled with a blood clot that is then remodeled to bone [14]. Our findings show, for the first time, that new bone formation in the extraction socket may be similar to the process of appositional bone formation [17]. This finding calls for reconsidering the current clinical understanding of socket healing and may be a key element to understanding how to treat sockets after tooth extraction. However, more research is needed to fully understand this observation.

Consequently, the current study found that two processes, the collapse and dimensional reduction in the socket and the appositional bone formation, lead together to the closure of the defect. This closure then resulted in cavitation formation in most of the cases observed in the study. It seems that the slow process of new bone formation within the socket is not able to close the defect alone; therefore, the body speeds up the process by means of socket shrinkage, enabling faster bone formation, at least in the occlusal part. Therefore, the regions that are not yet filled with new bone become cavities when the socket collapses, and the appositional bone from the defect walls comes into partial contact. In other words, it is possible that the body tries to turn a “critical size defect” of the socket that is not possible to undergo unassisted closure into a “non-critical size defect” by means of collapse, thus allowing the body to close the defect.

A search through the available literature revealed no previous radiological studies analyzing bone formation within the extraction sockets over different time points. However, other authors have described observations that were similar to the findings in this study. For example, a clinical study that analyzed models to evaluate dimensional change in the alveolar crest referred to “alveolar crest shifts” of 56–64% [18], which is similar to the inward movement and socket collapse observed in our study.

When looking carefully at the available literature, one can find similarities in the morphological descriptions. In particular, Misch and Judy described a classification system based on the tactile analog sense of the surgeon when drilling into the alveolar bone. Here, the D-qualities were classified as D1: dense cortical bone; D2: dense to porous cortical bone with coarse trabeculae; D3: thin cortical bone with fine central trabeculae; and D4: fine trabecular bone [19]. Moreover, various studies have shown a significant correlation between the surgeon’s tactile sense and CBCT data [18]. Therefore, tactile evaluation of the bone quality may resemble the described morphology in our study, containing the porous cortical quality in the occlusal part of the defect and the more “fine trabecular” part that is described as partially filled with new bone and partially occupied by cavitation.

The discrepancies between the results presented in this study and the previously observed and described findings in other preclinical and clinical studies may be understood when looking at the applied methods. Classical methods such as histological analysis are of great importance to understanding the biological process of new bone formation. However, they provide a one-dimensional analysis and mostly lack an overview perspective, especially when core biopsies are analyzed. Additionally, single time point radiological analyses that are normally performed to plan dental implants do not provide much information about the dimensional change over time. Therefore, the novelty of this radiological study lies in three aspects that enabled the observed findings—the three-dimensional analysis, the analysis over three time points (day 0, 3 months, and 6 months), and the radiological matching method used for analysis.

The limitations of the present study are mainly the number of analyzed subjects, the inclusion of only upper premolars, and the retrospective approach. Therefore, further studies are needed to analyze socket healing within other regions of the upper and lower jaw. Additionally, due to the retrospective approach and limitation to the available data the present study used two different groups of patients to analyze the dimensional change in the bone over time. In this context, a within-group analysis would have been more precise and should be considered in upcoming prospective studies.

Based on the findings presented here, it is possible to make several clinically relevant conclusions.

First, regarding the time point of implant insertion when sockets are left to heal unassisted, it should be considered that, based on our findings, the socket was not yet closed after 3 months. Therefore, this time point is not suitable for implant placement. This is encouraged by the statistically significantly higher new bone formation after 6 months. Therefore, authors recommend to choose a healing period of 6 months.

Second, this study showed different patterns of socket healing interindividual within the analyzed cohort, concluding that there is no guarantee that bone formation will occur after three months. Based on this, radiographic control rather than anecdotic assumptions must be performed before implantation, considering the lowest possible radiation dose burden for patients. Even though CBCT has a higher dose than that of the conventional OPG (approximate mean dose of 99.9 vs. 35.81 µSv, depending on the mode of view) [20], it has an excellent dose–image resolution ratio, with significantly more information regarding the clinical situation of the area of interest. Considering the ALARA principle [21], focused imaging, such as half-side or ROI-focused CBCT, should be applied. Further approaches have been investigated to achieve better dose reduction without the loss of information [22]. These recommendations were also found in various other studies in the literature [23,24]. Another noninvasive imaging approach is transalveolar ultrasound technology (TAU), which may provide comparable insights without additional exposure to ionizing radiation [25,26].

Third, our data showed that cavitation formation within the socket seems to be a physiological process that takes place in most cases and persists even after six months. These cavitations are mostly built by the collapse of the socket and the relatively fast new bone formation within the occlusal part. They are localized under a rather thin bone layer in the occlusal part of the socket and may have a critical role in the dental implantation process, including implant failures such as failing to reach adequate primary stability, lack of osseointegration during the healing period, or even infections during the oral retention period of the implants. These findings open novel topics that are of high interest for future research.

Finally, the outcomes of our study represent a paradigm shift and call for rethinking the present socket treatment strategies. When abandoning the theory that bone regeneration within the socket is achieved through remodeling of the initial blood clot [14], and considering the appositional process of bone formation observed in this study, it becomes clear that it is not logical to expect blood concentrates such as platelet-rich fibrin to become remodeled into bone once applied inside the extraction socket, or even to expect inflammatory cells of the peripheral blood to induce new bone formation. Therefore, a combination of a slowly degrading bone substitute material and blood concentrate seems to be a more promising strategy.

Additionally, this study showed premature closure of the occlusal part of the socket, along with socket collapse, resulted in the formation of cavities. In this case, it seems as if the premature closure of the socket is not an effective strategy to regenerate bone within the defect center, especially when taking into account that primary closure was shown to have no effect on bone formation in previous studies [27,28]. Instead, novel attempts to optimize bone regeneration within the socket, such as the recently presented Guided Open Wound Healing Concept, may be groundbreaking. In this concept, a polytetrafluoroethylene (PTFE) membrane is used in combination with bone substitute material to allow for socket reconstruction while respecting the anatomy of the alveolar ridge and without flap raising [29]. Ongoing studies will provide more data about the clinical efficacy of this concept and establish reproducible treatment protocols.

## 5. Conclusions

The present retrospective study analyzed unassisted socket healing after tooth extraction in the upper premolar region by means of 3D radiology after 3 (*n* = 18) and 6 months (*n* = 18). The results showed a morphological change in the socket, indicating an inwards movement of the socket walls towards the defect center. The new bone formation inside the socket was first evident within the occlusal part of the socket. However, new bone formation in terms of bone apposition was observed along the socket walls and did not reach the defect center in most cases. The attempts by the body to close the defect with a combination of socket collapse and appositional bone formation led to the formation of cavitation inside the socket. The novel findings observed in this study present a new understanding of socket healing, considering it as a patient-specific process. The evidenced data open new avenues for socket treatment strategies based on the anatomical, biological, and morphological understanding of the healing process. Additionally, the CBCT data highlight the importance of performing 3D analysis for planning dental implants to ensure bone volume, bone regeneration, and cavitation formation inside the socket.

## Figures and Tables

**Table 1:** Demographic data of the included patients.

	3 Months Group	6 Months Group
Women	10	9
Men	8	9
Mean age	49.6	51.8
Number of one rooted teeth	10	12
Number of two rooted teeth	8	6

**Table 2:** The percentage of the measured parameters in different dimensions.

Occlusal Height Loss in Bucco-Lingual View				
Healing period	Non-Classified Area (%)	New Bone Formation (%)	Collapse (%)	*n*
3 months	41.6 ± 20.6	39.1 ± 13.9	19.2 ± 9.2	18
6 months	29.6 ± 13.9	49.6 ± 6.7	20.6 ± 9.2	18
significance	Yes (*p* < 0.01)	No	No	
Occlusal Height Loss in Mesio-Distal View				
Healing period	Non-Classified Area (%)	New Bone Formation (%)	Collapse (%)	*n*
3 months	29.3 ± 18.8	39.7 ± 22.4	30.1 ± 9.0	18
6 months	15.7 ± 8.3	50.0 ± 10.8	34.3 ± 6.7	18
significance	Yes (*p* < 0.05)	No	No	
Lateral Bone Loss in Occlusal View				
Healing period	Non-Classified Area (%)	New Bone Formation (%)	Collapse (%)	*n*
3 months	21.4 ± 16.5	50.1 ± 6.9	28.0 ± 11.8	18
6 months	7.3 ± 4.9	64.3 ± 10.0	28.3 ± 8.9	18
significance	Yes (*p* < 0.001)	Yes (*p* < 0.001)	No	

**Table 3:** The buccal and lingual collapse on 3 and 6 months after tooth extraction.

Healing Period	Lingual Collapse (%)	Buccal Collapse (%)	Significance
3 months	11.0 ± 6.1	47.7 ± 12.3	Yes (*p* < 0.01)
6 months	24.0 ± 6.8	55.7 ± 29.1	No (*p* < 0.001)

## Data Availability

The raw data supporting the conclusions of this article will be made available by the corresponding author [S.G.] on request.
